# Fast dynamics perturbation analysis for prediction of protein functional sites

**DOI:** 10.1186/1472-6807-8-5

**Published:** 2008-01-30

**Authors:** Dengming Ming, Judith D Cohn, Michael E Wall

**Affiliations:** 1 Computer, Computational, and Statistical Scienes Division, Los Alamos National Laboratory, Los Alamos, New Mexico, USA; 2 School of Life Sciences, Nanjing University, Nanjing, Jiangsu Province, China; 3 Bioscience Division, Los Alamos National Laboratory, Los Alamos, New Mexico, USA; 4 Center for Nonlinear Studies, Los Alamos National Laboratory, Los Alamos, New Mexico, USA

## Abstract

**Background:**

We present a fast version of the dynamics perturbation analysis (DPA) algorithm to predict functional sites in protein structures. The original DPA algorithm finds regions in proteins where interactions cause a large change in the protein conformational distribution, as measured using the relative entropy *D*_**x**_. Such regions are associated with functional sites.

**Results:**

The Fast DPA algorithm, which accelerates DPA calculations, is motivated by an empirical observation that *D*_**x **_in a normal-modes model is highly correlated with an entropic term that only depends on the eigenvalues of the normal modes. The eigenvalues are accurately estimated using first-order perturbation theory, resulting in a *N*-fold reduction in the overall computational requirements of the algorithm, where *N *is the number of residues in the protein. The performance of the original and Fast DPA algorithms was compared using protein structures from a standard small-molecule docking test set. For nominal implementations of each algorithm, top-ranked Fast DPA predictions overlapped the true binding site 94% of the time, compared to 87% of the time for original DPA. In addition, per-protein recall statistics (fraction of binding-site residues that are among predicted residues) were slightly better for Fast DPA. On the other hand, per-protein precision statistics (fraction of predicted residues that are among binding-site residues) were slightly better using original DPA. Overall, the performance of Fast DPA in predicting ligand-binding-site residues was comparable to that of the original DPA algorithm.

**Conclusion:**

Compared to the original DPA algorithm, the decreased run time with comparable performance makes Fast DPA well-suited for implementation on a web server and for high-throughput analysis.

## Background

Prediction of protein functional sites is a key aspect of protein function prediction [1], and can be an important step in identifying small-molecule interactions for drug discovery [2]. It can also potentially be used as a pre-processing step to reduce the search space in computational docking algorithms. There are many methods to predict functional sites–here we emphasize those that make use of analysis of protein structure and dynamics. Existing protein structure analysis methods are based on diverse principles, including: association of functional sites with surface clefts that have extreme values of volume [3-6] or other shape descriptors [7-11]; identifying spatial clusters of methyl probes that exhibit energetically favorable interactions with the protein [12]; association of functional sites with charged surface residues either in unfavorable electrostatic environments [13] or with anomalous predicted pH titration curves [14]; identifying spatial clusters of residues whose diversity appears to be correlated with changes in protein function [15,16]; defining structural features (e.g. motifs) associated with functional sites [17-22]; identifying residues that are on average close to other residues in the protein (closeness centrality) [23-25]; and machine-learning prediction of functional sites/residues using sequence, structure, and chemical features from training sets [26-28]. Principles of methods that consider protein dynamics include association of functional sites with: hinge regions [29,30]; regions where the harmonic vibrations are largely determined by high-frequency modes [31]; intrinsically disordered regions that are highly mobile in the absence of a molecular interaction partner [32]; and residues where mutations cause a large change in the couplings of local perturbations to remote, local changes in the distribution of folded vs. unfolded states of the protein [33]. Information from complementary methods may be integrated for functional site prediction [34,35].

We recently developed an additional approach to prediction of protein functional sites that is based on analysis of protein dynamics [36-39]. To help motivate the approach, we note that cellular functions are regulated by molecular interactions that alter protein activity. To enable such control, protein activity, and therefore protein conformational distributions, must be susceptible to alteration by molecular interactions at functional sites. In other words, protein activity should be controllable by allosteric effects (allostery).

Weber [40] recognized the importance of considering changes in the *full conformational distribution *to understand allostery, as opposed to considering mechanistic changes among *discrete, well-defined structural states *in earlier models due to Monod, Wyman, and Changeux [41]; and Koshland, Nemethy, and Filmer [42]. Weber's perspective is well-aligned with more recent emphases on the need to consider allostery from a global thermodynamic/statistical perspective [43,36-39,33,45]. It is also well-aligned with modern rate theories based on the control of protein activity by dynamical transitions among conformational substates [46], as originally suggested by spectroscopic assays of ligand-binding at low-temperature [47,48].

Given the above considerations, we hypothesized that protein functional sites might tend to evolve at control points where interactions cause a large change in the protein conformational distribution [36]. To test this hypothesis, we developed a method called dynamics perturbation analysis (DPA) to quantify changes in protein conformational distributions due to molecular interactions [36,37], examined 305 protein structures from the GOLD [49] docking test set [38], and found that interactions at small-molecule binding sites cause a relatively large change in protein vibrations.

Motivated by these results, we developed a DPA-based algorithm that successfully predicts small-molecule binding sites at locations where interactions cause a large change in protein vibrations [38]. This method was evaluated in Ref. [38] using 305 proteins in the GOLD [49] docking test set of protein-ligand structures. For the test, only the top-ranked functional site was selected and was used to predict the location of the ligand-binding site. This is a relatively strict requirement; in other published methods for predicting functional sites [11], performance often is evaluated by allowing for any of several predicted functional sites to overlap a known ligand-binding site. The method produced at least one predicted functional site for 287 of the 305 proteins in the test set. In 87% of cases (250 proteins), at least one predicted residue was in the ligand-binding site. The recall of binding-site residues (percentage of binding-site residues found among the predicted residues) was at least 30% for 80% of cases, and was at least 50% for 76% of the cases. The precision of the predicted residues (percentage of predicted residues found among the binding-site residues) was at least 30% for 68% of the cases, and was at least 50% for 44% of the cases. The statistical significance of the overlaps was assessed using a null model in which surface residues were randomly selected. Using the null model, a P-value was calculated to evaluate predictions for the 250 proteins in which at least one predicted residue was in the ligand-binding site. The P-value estimated the probability of obtaining a precision at least as high as the observed precision by randomly selecting surface residues [38]. For 87% of the cases, the P-value was 10^-3 ^or smaller, indicating a statistically significant overlap. The performance of the DPA method compared favorably to that of a cleft analysis method for predicting ligand-binding residues.

The original DPA algorithm is a highly innovative approach that performs well. However, the computational requirements limit the utility of the original method. For example, it takes about an hour to analyze a 150-residue protein domain using DPA, and the method doesn't scale well to larger systems. Here, we report an improved algorithm based on use of first-order perturbation theory that will facilitate the use of DPA in high-throughput scenarios and increase its utility, e.g., for web server applications. The algorithm, called Fast DPA, enables a dramatic decrease in the time required to predict protein functional sites, with performance that is comparable to the original DPA algorithm.

## Methods

### Dynamics perturbation analysis

**Figure 1:**
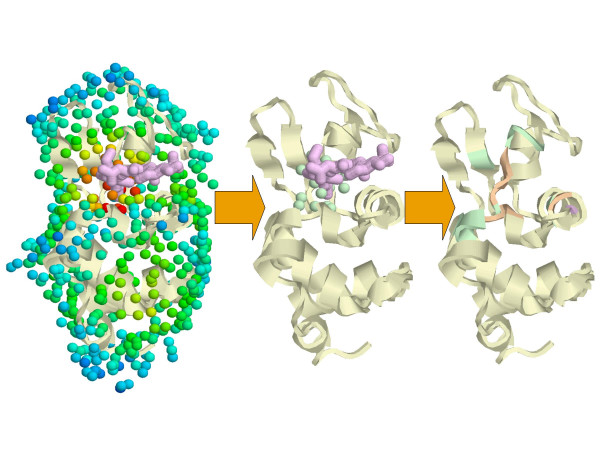
Application of Dynamics Perturbation Analysis (DPA) to predict protein functional sites. *Left*. In this example, the surface of lysozyme (PDB entry 1JEF [50], yellow cartoon) is decorated with test points (533 spheres at a density of 1 point per Å^2^), and the degree to which the test points individually perturb the protein conformational distribution is calculated (temperature-coded coloring of the spheres). A tri-NAG molecule (purple wireframe) binds in the active site. Warm-colored spheres indicate where the perturbation is large. *Center*. Points where the perturbation is largest are selected and clustered (green spheres). *Right*. C_α _atoms within 6 Å of the DPA cluster are selected, and the associated residues define the predicted functional site (16 residues). For comparison, C_α _atoms within 6 Å of the tri-NAG are selected; we use the associated residues to define the actual functional site (7 residues). The overlapping residues (6 residues) are shown in orange; there are 10 predicted residues that do not exactly match the functional site (green), and there is 1 functional site residue that is not among the predicted residues (purple, in the helix on the right hand side).

Our overall approach for predicting functional sites is based on a method called dynamics perturbation analysis (DPA) [36,38,37]. In DPA, a protein is decorated with *M *surface points that interact with neighboring protein atoms, as illustrated for Protein Data Bank entry 1JEF [50] in Fig. 1. The protein conformational distribution *P*(**x**) is calculated in the absence of any surface points, and *M *protein conformational distributions *P*^(*m*)^(**x**) are calculated for the protein interacting with each point *m*. The conformational distributions are calculated using a coarse-grained model of molecular vibrations, and the distributions *P*^(*m*)^(**x**) are calculated from models of the protein in complex with each surface point. The relative entropy, or Kullback-Leibler divergence [51], $D_{x}^{\left(m\right)}$ between *P*(**x**) and *P*^(*m*)^(**x**) is calculated for each point *m*, and is used as a measure of the change in the protein conformational distribution upon interacting with point *m*:

$$D_{x}^{\left(m\right)}=\int d^{3N}xP^{\left(m\right)}\left(x\right)\ln\frac{P^{\left(m\right)}\left(x\right)}{P\left(x\right)}$$

In the present case (unlike in other useful biological applications [52-56]), the relative entropy is not just an *ad hoc *measure; rather, it has real biophysical significance [39,57]: $k_{B}TD_{x}^{\left(m\right)}$, where *T *is the temperature and *k*_*B *_is Boltzmann's constant, is the free energy required to change the protein conformational distribution from an equilibrium distribution *P*(**x**) to a non-equilibrium distribution *P*^(*m*)^(**x**).

Thus far, DPA calculations have most often been performed using a simple model of protein vibrations–the elastic network model (ENM) [58-61]. In the ENM, C_α _atoms are extracted from an atomic model of a protein, and an interaction network is generated by connecting springs between all atom pairs (*i, j*) separated by a distance less than or equal to a cutoff distance *r*_c_. Each spring has the same force constant *γ*, is aligned with the separation between the connected atoms, and has an equilibrium length equal to the distance *d*_*ij *_between the atoms in the initial model. Thus, the potential energy is given by $U\left(x\right)=\gamma /2\sum_{i>j}\varepsilon_{ij}{\left(\left|x_{i}-x_{j}\right|-d_{ij}\right)}^{2}$, where *ε*_*ij *_= 1 if atoms *i *and *j *are connected, and *ε*_*ij *_= 0 otherwise. The interaction between the protein and a surface point *m *is modeled by connecting springs of force constant *γ*_s _between the surface point and all protein atoms within a cutoff distance *r*_s _of the surface point. The protein coordinates are not modified in modeling the interaction. The dynamics are defined using normal mode analysis of the model. In this model, the reference distribution *P*(**x**) is given by

P(x)=∏i=13N,λi≠0(λi2πkBT)12e−12kBTλi|(x−x0)⋅vi|2

In Eq. (2), *N *is the number of atoms in the protein; **x**_0 _is the equilibrium structure; and *λ*_*i *_and **v**_*i *_are the *i*^th ^eigenvalue and eigenvector of the Hessian ${H:h_{ij}=\partial U/\partial x_{i}\partial x_{j}|}_{x_{0}}$. The perturbed distribution *P*^(*m*)^(**x**) is similar to Eq. (2), but substituting the eigenvalues and eigenvectors ${\bar{\lambda }}_{i}^{\left(m\right)}$ and ${\bar{v}}_{i}^{\left(m\right)}$ of the pseudo-Hessian ${\bar{H}}^{\left(m\right)}$ for *λ*_*i *_and **v**_*i*_. ${\bar{H}}^{\left(m\right)}$ is derived from the full Hessian **H**^(*m*) ^for the protein model in the presence of the surface point *m*:

$$H^{\left(m\right)}=\left(\begin{array}{cc} H_{P}^{\left(m\right)} & G^{\left(m\right)}\\ G^{{\left(m\right)}^{T}} & H_{S}^{\left(m\right)} \end{array}\right).$$

The sub-matrix $H_{P}^{\left(m\right)}$ couples the protein coordinates, the sub-matrix $H_{S}^{\left(m\right)}$ couples the test-point coordinates, and the submatrix **G**^(*m*) ^couples the protein to the test point. In terms of these matrices, ${\bar{H}}^{\left(m\right)}$ is given by [37]

$${\bar{H}}^{\left(m\right)}=H_{P}^{\left(m\right)}-G^{\left(m\right)}H_{S}^{{\left(m\right)}^{-1}}G^{{\left(m\right)}^{T}}.$$

Using expressions for *P*(**x**) and *P*^(*m*)^(**x**), Eq. (1) becomes [36,37]

$$D_{x}^{\left(m\right)}=\frac{1}{2}\sum_{i=7}^{3N}\left(\log\frac{{\bar{\lambda }}_{i}^{\left(m\right)}}{\lambda_{i}}+\sum_{j=7}^{3N}\frac{\lambda_{j}}{{\bar{\lambda }}_{i}^{\left(m\right)}}{\left|{\bar{v}}_{i}^{\left(m\right)}\cdot v_{j}\right|}^{2}-1\right).$$

The first six modes involve zero eigenvalues and are ignored in the sums. Equation (5) is the central equation that enables DPA.

**Figure 2:**
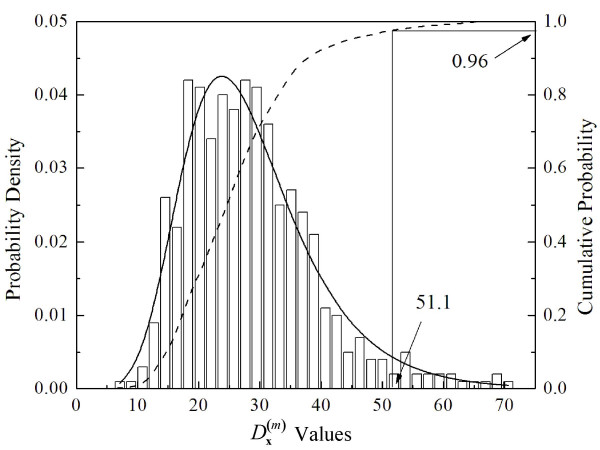
Distribution of $D_{x}^{\left(m\right)}$ values for 4859 points on the surface of lysozyme 1JEF (the number of points was increased in this case to evaluate the fit). The distribution is well-fit by an extreme value distribution (Eq. (6)) with parameters *μ *= 23.07 and *β *= 8.45 (solid line). By examining the cumulative distribution (dashed line), the fit is used to find surface points that lie within the upper 96% of the distribution; these points are used to predict functional sites.

To use DPA to predict functional sites, we make use of the fact that, empirically, the distribution of *y *= $D_{x}^{\left(m\right)}$ values on the surface of a protein calculated using Eq. (5) is observed to obey an extreme value distribution (Fig. 2),

$$\rho \left(y\right)=\frac{1}{\beta }e^{\frac{y-\mu }{\beta }-e^{\frac{y-\mu }{\beta }}}.$$

First, DPA is performed on a protein and the distribution of $D_{x}^{\left(m\right)}$ values is modeled using Eq. (6). Points with $D_{x}^{\left(m\right)}$ values in the upper 96% of the modeled distribution are selected and are spatially clustered. The clusters are ranked according to the mean value of $D_{x}^{\left(m\right)}$ within the cluster, and all clusters are considered to be potentially associated with a functional site. Finally, residues in the neighborhood of the clusters are selected and form the basis for functional site predictions.

### Fast dynamics perturbation analysis

Fast DPA is based on a simple empirical observation: for dynamics defined by normal modes, the total value of *D*_**x **_in Eq. (5) is highly correlated with just the first (entropic) term,

$$D_{x}^{\lambda ,(m)}=\frac{1}{2}\sum_{i=7}^{3N}\log\frac{{\bar{\lambda }}_{i}^{\left(m\right)}}{\lambda_{i}}.$$

Hereafter we refer to $D_{x}^{\lambda ,(m)}$ simply as $D_{x}^{\lambda }$. Observation of this correlation motivates the use of $D_{x}^{\lambda }$ as a surrogate for *D*_**x **_in DPA, and, because $D_{x}^{\lambda }$ only involves eigenvalues, creates an avenue for accelerating DPA. The acceleration arises because the eigenvalues of the normal models of the protein in the presence of test points are well-approximated using first order perturbation theory. In this approximation, the pseudo-Hessian ${\bar{H}}^{\left(m\right)}$ of the protein in the presence of point *m *is written as the Hessian **H **of the protein in the absence of the ligand plus a perturbation term *δ*${\bar{H}}^{\left(m\right)}$:

$${\bar{H}}^{\left(m\right)}=H+\delta {\bar{H}}^{\left(m\right)},$$

where the expression for ${\bar{H}}^{\left(m\right)}$ is as in previous studies [37,38]. To estimate the eigenvalues of ${\bar{H}}^{\left(m\right)}$, we use the canonical first-order perturbation theory expression,

$$\lambda_{i}^{\left(m\right)}\approx \lambda_{i}+v_{i}^{T}\delta {\bar{H}}^{\left(m\right)}v_{i},$$

where *λ*_*i *_is the *i*th eigenvalue of **H**.

The Fast DPA algorithm is the same as the original DPA algorithm, except instead of using values of *D*_**x**_, the analysis is based on values of $D_{x}^{\lambda }$ estimated using perturbation theory. (It is possible to evaluate all terms in Eq. (5) using first-order perturbation theory, but doing so would not accelerate the method because the computational cost is comparable to that of solving the full eigenvalue problem in original DPA.)

### Implementation of Fast DPA

Our implementation of DPA and Fast DPA here follows our previous implementation of DPA for functional site prediction [38]. Given an input PDB structure, MSMS [62] was run with a 1.5 Å probe radius and a triangulation density of 1 vertex per Å^2 ^to generate test points on the surface of the protein. As when using original DPA to predict functional sites, perturbations were calculated using every other point in the MSMS output (we also tried using every point, but this led to decreased performance in the precision measures). The cutoff *r*_*c *_for interactions between protein C_α _atoms was 8.5 Å. For some proteins, this cutoff yielded more than six zero-frequency modes, indicating that the network of springs was too sparse (for example, if only one spring connects two domains, then free rotations about the spring yield two additional zero-frequency modes). In these cases, the connectivity of the elastic network model was increased by incrementing *r*_*c *_in 1 Å steps until the additional zero-frequency modes were eliminated. The cutoff *r*_*s *_for interactions between a test point and the protein was 14 Å, and the interaction strength between a test point and protein atoms was *γ*_*s *_= 12*γ*, or 12 times the strength of the interaction between two protein atoms. Results are independent of the value of *γ*.

### Implementation of functional site prediction using DPA

To predict functional sites, the distribution of *y *= $D_{x}^{\left(m\right)}$ values was fit using Eq. (6). Points with $D_{x}^{\left(m\right)}$ values in the upper 96% of the distribution were selected and spatially clustered using the OPTICS algorithm [63] with a distance threshold of 6 Å and a minimum of 3 points per cluster. C_α _atoms within 6 Å of any point in a cluster were selected and were used to define predicted functional sites. The sites were ranked according to the mean value of $D_{x}^{\left(m\right)}$ within the corresponding cluster of points. Only the top-ranked predicted site was used for the evaluation of performance described below.

## Results and Discussion

### Results that motivate Fast DPA

**Figure 3:**
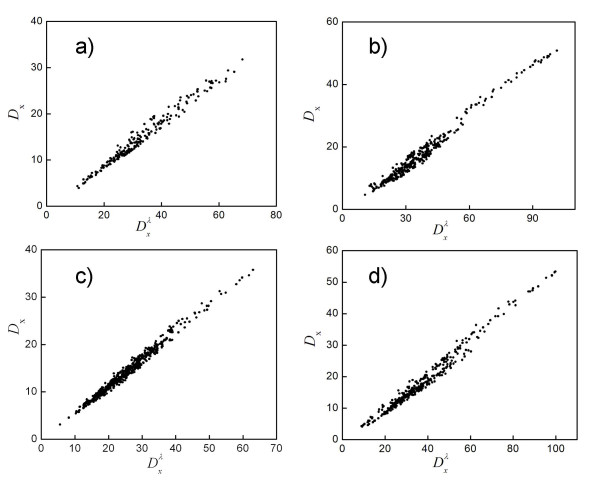
Values of *D*_x _(y-axis) and $D_{x}^{\lambda }$ (x-axis) calculated using original DPA are plotted for four PDB entries (values of the Pearson correlation, *C*, between the two, are listed here parenthetically): a) 1AEC [65], from an actinidin-E-64 complex (*C *= 0.988); b) 1FKI [66], from a FKBP complex (0.989); c) 1JEF [50], from a lysozyme complex (0.992); and d) 1STP [67], from a biotin complex (0.989).

To motivate the use of $D_{x}^{\lambda }$ instead of *D*_**x **_for DPA, we analyzed proteins from the GOLD test set. We found that *D*_**x **_is highly correlated with $D_{x}^{\lambda }$ for these cases; Fig. 3 illustrates the agreement for four proteins. This is not a trivial result mathematically (see Eqs. (5) and (7))–it means that $\sum \log\left({\bar{\lambda }}_{i}^{\left(m\right)}/\lambda_{i}\right)$ is highly correlated with $\sum_{i}\sum_{j}{\left|{\bar{v}}_{i}^{\left(m\right)}\cdot v_{j}\right|}^{2}\lambda_{j}/{\bar{\lambda }}_{i}^{\left(m\right)}$.

**Figure 4:**
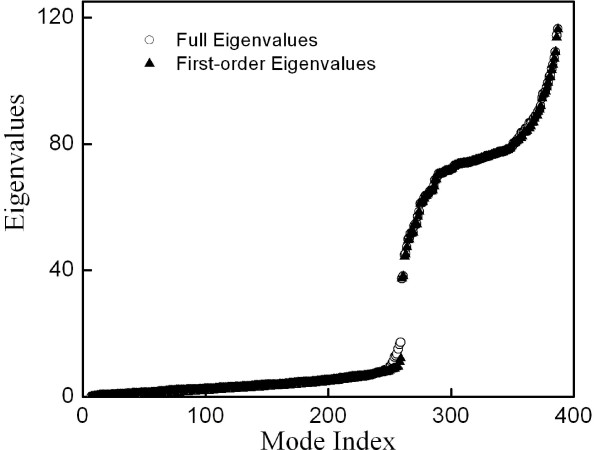
Eigenvalues (used for calculation of $D_{x}^{\lambda }$) that are estimated using perturbation theory (filled triangles) are a good approximation to the true eigenvalues of a lysozyme elastic network model (open circles).

**Figure 5:**
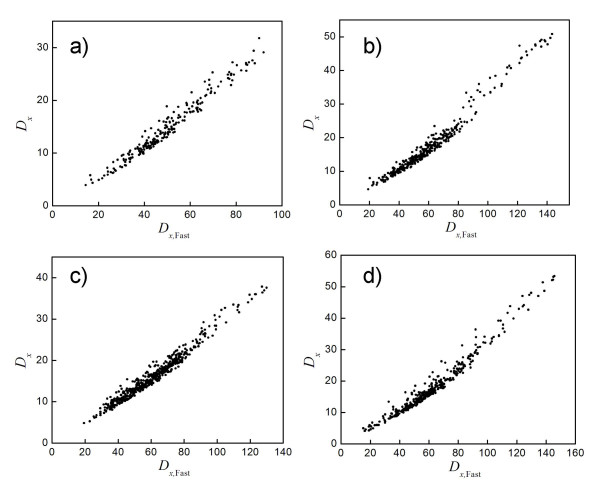
Values of *D*_x _calculated using original DPA (y-axis) and $D_{x}^{\lambda }$ calculated using Fast DPA (x-axis) are plotted for four PDB entries (values of the Pearson correlation between the two are listed here parenthetically): a) 1AEC (0.981); b) 1FKI (0.982); c) 1JEF (0.981); d) 1STP (0.980).

To motivate the use of perturbation theory to estimate $D_{x}^{\lambda }$, we compared the true eigenvalues to those estimated using perturbation theory for proteins in the GOLD test set. Because in our model the strength of the spring that connects the test points to the protein is 12 times the strength of the spring that connects protein atoms to each other (Methods), it was not obvious that first-order perturbation theory would yield reasonable estimates of eigenvalues. However, we had hoped for success based on the fact that we were only adding a single test point to the model, compared to, typically, O(100) protein C_α _atoms. As illustrated for lysozyme in Fig. 4, we did find that Eq. (9) approximates well the true eigenvalues obtained by diagonalization of **H**^(*m*)^. Finally, we found that *D*_**x **_calculated using original DPA was highly correlated with $D_{x}^{\lambda }$ calculated using Fast DPA, as illustrated for four proteins in Fig. 5.

### Evaluation of Fast DPA for prediction of functional sites

**Figure 6:**
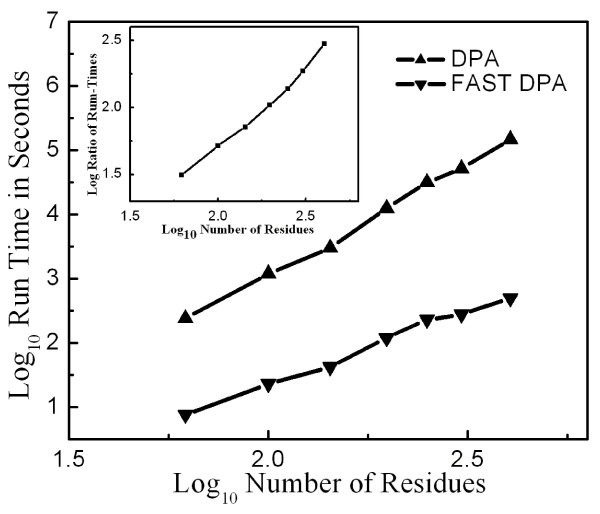
Comparison of run times for DPA (upwards-pointing triangles) vs. Fast DPA (downwards-pointing triangles) for various protein sizes. The inset shows the ratio of run times for various protein sizes.

The above results motivated us to develop the Fast DPA algorithm for prediction of protein functional sites (Methods). Through use of first-order perturbation theory, Fast DPA replaces matrix diagonalization by matrix-vector multiplication for each test point (Eq. (9)). Because matrix diagonalization requires O(*N*^3^) operations, and matrix-vector multiplication requires O(*N*^2^) operations, we expected Fast DPA to run *N*-fold faster than the original DPA. We found this to be the case (Fig. 6): the original DPA scales roughly as *N*^3.45^, while fast DPA scales roughly as *N*^2.29^, yielding a factor of *N*^1.16 ^decrease in the time required to perform Fast DPA vs. DPA (here, *N *is the number of residues in the protein).

Because *D*_**x **_calculated using original DPA and $D_{x}^{\lambda }$ calculated using Fast DPA are highly correlated (Fig. 5), we expected the performance of Fast DPA in predicting functional site residues to be comparable to that of the original DPA. We analyzed the performance of the algorithm on the 305-protein GOLD [49] test set, which was used to evaluate the original DPA algorithm [38]. Each prediction has an associated recall (fraction of residues in the binding site that are among those in the rank-1 prediction) and precision (fraction of rank-1 predicted residues that are among those in the binding site). To evaluate performance statistically, we use (1) the fraction of binding sites for which the recall is greater than or equal to a minimum value, and (2) the fraction of fraction of rank-1 predictions for which the precision is greater than or equal to a minimum value.

**Figure 7:**
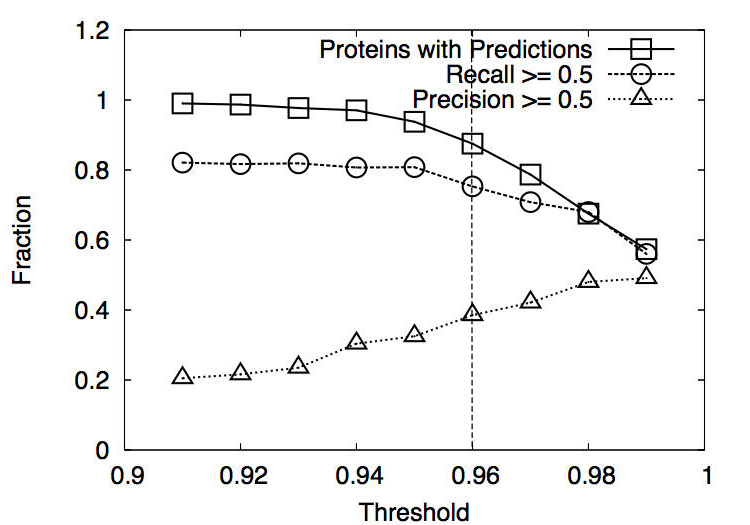
Comparison of Fast DPA performance using different thresholds of the extreme value distribution (Eq. (6)). The y-axis is either the fraction of proteins for which a prediction is made (squares), the fraction of binding sites with a recall of at least 0.5 (circles), or the fraction of predictions with a precision of at least 0.5 (triangles). The threshold is indicated on the x-axis; the 0.96 threshold used for Figs. 9 and 10 is indicated using a vertical dashed line.

**Figure 8:**
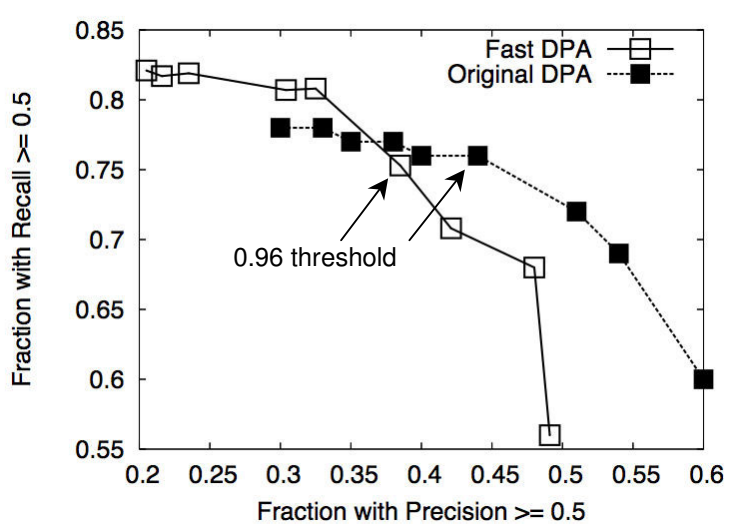
Comparison of Fast DPA vs. original DPA precision and recall statistics at different thresholds of the extreme value distribution (Eq. (6)). The curves are similar to precision-recall curves: the y-axis is the fraction of binding sites with a recall of at least 0.5, and the x-axis is the fraction of binding sites with a precision of at least 0.5. Fast DPA values are indicated using open squares, and original DPA is indicated using filled squares. Points corresponding to a threshold of 0.96 are indicated using arrows.

Figure 7 compares the performance of Fast DPA using different thresholds of the extreme value distribution, and is equivalent to Fig. 8 in [38]. The nominal threshold of 0.96 indicated in this figure is equivalent to that chosen for original DPA. Fig. 8 compares the performance of Fast DPA with original DPA for different thresholds. When the threshold is 0.96 or smaller, the recall statistics of Fast DPA tend to be better, and the precision statistics of original DPA tend to be better. When the threshold is 0.97 or higher, original DPA outperforms Fast DPA in both precision and recall statistics.

**Figure 9:**
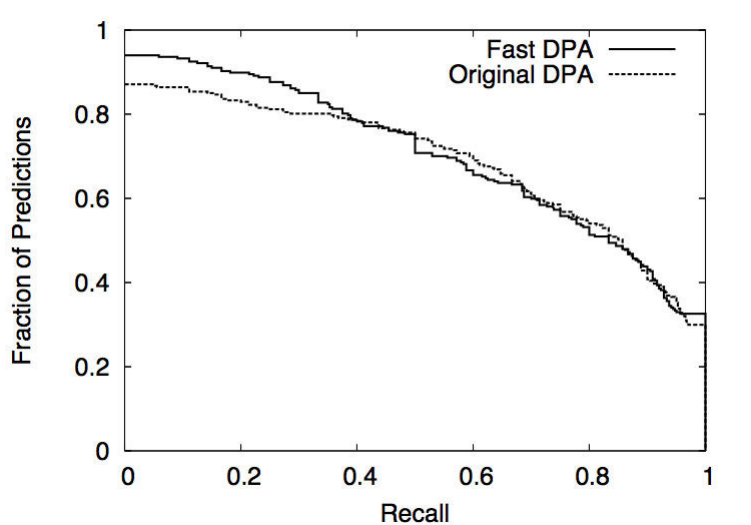
Comparison of recall of binding-site residues using DPA vs. Fast DPA for 287 (number of predictions using DPA) or 267 (number of predictions using Fast DPA) proteins in the 305-protein GOLD test set. The y-axis indicates the fraction of proteins with a recall at least as high as the value on the x-axis (y-values should be read from the top of each step).

**Figure 10:**
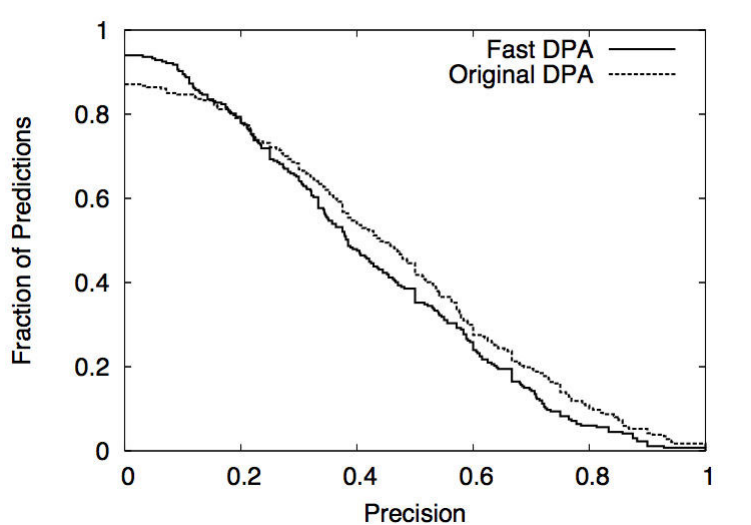
Comparison of precision of predicted residues using DPA vs. Fast DPA (see also Fig. 9). The y-axis indicates the fraction of proteins with a precision at least as high as the value on the x-axis (y-values should be read from the top of each step).

At the nominal threshold value of 0.96, the performance of Fast DPA is comparable to that of original DPA. At this threshold, original DPA yielded 287 rank-1 predictions for the test set (rate of 94%), whereas Fast DPA yielded 267 rank-1 predictions (rate of 86%) (Table 1). However, Fast DPA makes 251 predictions that have at least one residue that overlaps the binding site, while original DPA makes 250 such predictions, yielding a higher rate of locating binding sites for rank-1 Fast DPA predictions (94%) than for original DPA (87%) (Table 1). The recall statistics tend to be a bit better for Fast DPA (Table 1, Fig. 9), and the precision statistics tend to be better for original DPA (Table 1, Fig. 10).

**Table 1:** Performance statistics for Fast DPA and original DPA using a threshold of 0.96

	Rank-1 Predictions^a^	Any match^b^	Recall ≥ 0.3^c^	Precision ≥ 0.3^d^	Recall ≥ 0.5^c^	Precision ≥ 0.5^d^
Original	287	0.87	0.80	0.68	0.76	0.44
Fast	267	0.94	0.86	0.65	0.75	0.38

^a ^Number of proteins for which at least one DPA cluster was produced, out of 305 total.

^b ^Fraction of rank-1 predictions that have at least one overlapping residue with the binding site.

^c ^Fraction of binding sites for which the recall was at least 0.3 or 0.5.

^d ^Fraction of predictions for which the precision was at least 0.3 or 0.5.

## Conclusion

Use of Fast DPA enables functional site predictions to be performed *N*-fold faster than original DPA, with comparable performance in predicting residues in functional sites. The acceleration will facilitate optimization of Fast DPA for functional site predictions. Calculations that once took hours using DPA now may be performed in a matter of minutes, making practical the use of DPA via a web server. Indeed, high-throughput analysis using Fast DPA has already produced over 60,000 predicted functional sites for about 50,000 protein domains in the SCOP [64] database (J.D. Cohn, D. Ming, and M.E. Wall, in preparation). These predictions will provide a rich source of information for developing hypotheses concerning mechanisms of protein function.

## Authors' contributions

DM implemented the Fast DPA algorithm, tested its performance, and helped to draft the manuscript. JC provided assistance with databases and automation. MW conceived of the study, coordinated the work, and drafted the manuscript. All authors read and approved the final manuscript.
